# Lymphatic Filariasis: A Systematic Review on Morbidity and Its Repercussions in Countries in the Americas

**DOI:** 10.3390/ijerph19010316

**Published:** 2021-12-28

**Authors:** Zulma M. Medeiros, Amanda V. B. Vieira, Amanda T. Xavier, Gilberto S. N. Bezerra, Maria de Fátima C. Lopes, Cristine V. Bonfim, Ana M. Aguiar-Santos

**Affiliations:** 1 Fundação Oswaldo Cruz, Departamento de Parasitologia, Instituto Aggeu Magalhães, Recife 50670-420, Brazil; zulma.medeiros@fiocruz.br (Z.M.M.); amanda-xavier@hotmail.com (A.T.X.); anamaria.aguiar@fiocruz.br (A.M.A.-S.); 2 Programa de Pós-Graduação em Ciências da Saúde, Universidade de Pernambuco, Recife 50100-130, Brazil; 3 Materials Research Institute, Technological University of the Shannon: Midlands Midwest, N37 HD68 Athlone, Ireland; a00278630@student.ait.ie; 4 Ministério da Saúde, Secretaria de Vigilância em Saúde, Brasília 70723-040, Brazil; fatimacostalopes@gmail.com; 5 Ministério da Educação, Fundação Joaquim Nabuco, Diretoria de Pesquisas Sociais, Recife 52061-540, Brazil; cristine.bonfim@uol.com.br; 6 Programa de Pós-Graduação em Saúde Coletiva, Universidade Federal de Pernambuco, Recife 50670-901, Brazil

**Keywords:** lymphatic filariasis, morbidity, hydrocele, lymph scrotum, lymphoedema, acute dermatolymphangioadenitis, elephantiasis

## Abstract

The Global Program to Eliminate Lymphatic Filariasis (GPELF) is a program that aims to eliminate lymphatic filariasis by 2030. The GPELF strategy is based on interrupting transmission using mass drug administration (MDA) and, in parallel, managing morbidity cases. However, it has been seen that there is a shortage of research in the literature and public policies regarding this last pillar. In this study, we reviewed the literature and available information regarding the burden of filarial morbidity. In addition, we identified that in the Americas, the implementation of structured services with regard to morbidity assistance in the Americas was scarce. We formed a review that aimed to assess the pathogenesis, epidemiology, repercussions, and treatment of filarial morbidity in countries in the Americas where lymphatic filariasis is endemic. Structured searches were carried out on PubMed, LILACS, Scopus, and Web of Science databases without time and language restrictions. Three reviewers evaluated the 2150 studies and performed data extraction, and quality assessment by assigning scores to the studies found. The current literature and available information on the burden of filarial morbidity, as well as the implementation of structured services with regard to morbidity assistance in the Americas, were all found to be scarce. Now that this knowledge gap has been identified, both health services and researchers need to seek the implementation and enhancement of the maintenance of GPELF strategies that relate to the morbidity pillar.

## 1. Introduction

Lymphatic filariasis (LF) is a neglected tropical disease caused by *Brugia malayi*, *Brugia timori*, and *Wuchereria bancrofti* and over 90% of cases are caused by the last. These parasites are transmitted via a number of different mosquito hosts, which vary geographically. LF is considered endemic in 72 countries, and Brazil, the Dominican Republic, Guyana, and Haiti are the four remaining countries in the Americas where it is considered endemic [1]. The World Health Organization (WHO) classified this parasitic infection as the second most common cause of long-term disability after mental illness [2].

In 2000, the WHO launched the Global Program to Eliminate Lymphatic Filariasis (GPELF) with the aim of eliminating LF by 2030. The strategy of the GPELF is based on interrupting transmission using mass drug administration (MDA), and, in parallel, alleviating and preventing suffering and disability in those who already have chronic manifestations of the disease [3,4].

Morbidity in LF is expressed as painful and profoundly disfiguring visible chronic manifestations that include lymphoedema (acute dermatolymphangioadenitis—ADLA and elephantiasis) and male urogenital disease (hydrocele and lymph scrotum). ADLA bacterial infections cause significant pain and fever, and also occur in phases. Other less commonly reported clinical expressions include lymphoedema of the breast, swelling of the vulva, and rheumatic and respiratory problems [3].

The MDA program is advanced, and many endemic countries have introduced the strategy and achieved tremendous success, thus contributing to reducing global LF infection rates from 120 million individuals in 1997 to 56 million individuals in 2017 [4]. However, morbidity management remains less widespread and unsuccessful, and the literature is scarcer, and less information is available regarding the implementation of programs that aim to address this pillar [5].

The objectives of this review were to analyze the main findings regarding the pathogenesis, epidemiology, repercussions, and treatment of filarial morbidity that have been adopted in countries in the Americas where lymphatic filariasis is endemic, in order to support the progress of the GPELF with regard to the pillar of morbidity.

## 2. Materials and Methods

In this systematic review, we adhered to the PRISMA 2020 guidelines and checklist—Preferred Reporting Items for System Reviews and Meta-Analyses [6] to reduce the possibility of inserting bias.

### 2.1. Data Sources and Search Strategy

A bibliographic survey was carried out by looking for scientific articles from May 1987 to July 2021, without restrictions with regard to the year of publication and language. Searches were performed in national and international databases (LILACS (Latin American and Caribbean Literature on Health Sciences), PubMed (National Center Biotechnology Information), Scopus (Elsevier), and Web of Science (Clarivate Analysis)).

For this, the following search-key was used: (((Elephantiasis, Filarial) OR (“lymphatic filariasis” OR filaria *)) AND ((m *) OR (elephantias * OR hydrocel * OR (lymphoedema OR lymphoedema) OR lymphadenopathy * OR (“Acute dermatolymphangioadenit *” OR ADLA) OR lymphangit *))) AND (Brazil OR Guyana OR “Dominican Republic” OR Haiti OR “Trinidad and Tobago” OR “Costa Rica” OR Suriname).

### 2.2. Study Selection and Data Extraction

Initially, the articles were selected independently by 2 reviewers (ATX and AVBV). Before the screening, duplicate studies were removed, and the remaining studies passed through to the first selection stage (reading of titles and abstracts). After this first screening, some studies were considered ineligible, and the remainder were sent to the next stage of the selection process (reading the full text). In this phase, in addition to data extraction, eligibility standards were validated once again. In the presence of any disagreement, a more experienced third reviewer (AMAS) made their assessment. The inclusion criteria for this review were as follows: (i) LF morbidity cases (male urogenital disease and lymphoedema); (ii) endemic countries and those that have already received LF elimination verification in the Americas. Articles were excluded if (i) they did not agree with the inclusion criteria and addressed cases of filarial morbidity in locations other than the Americas; (ii) they were narrative, systematic literature reviews with or without meta-analyses; (iii) book chapters; (iv) conference publications; (v) letters to editors; (vi) case reports, and (vii) studies that do not address morbidities.

From the analysis of the inclusion criteria, the following data were extracted from the studies: (a) author (s), (b) country, (c) kind of study, (d) population, (e) objective, (f) number of morbidity cases, and (g) outcomes. Two reviewers (ATX and AVBV) performed this extraction independently and in the presence of any discrepancy, a more experienced third reviewer (AMAS) performed the data analysis. After the inclusion of selected articles from the databases, a manual search was performed in their reference lists, with the purpose of analyzing possible eligible studies that were not previously identified. This step was followed by the same protocols previously applied.

### 2.3. Risk of Bias and Quality Assessment

The quality assessment of these articles was performed by 2 independent reviewers (ATX and AVBV) using the Standard Quality Assessment Criteria for Assessing Primary Research Articles from a Variety of Fields [7]. It consists of a qualitative and quantitative analysis instrument, which examines the methodological quality of each study included in the review. Each evaluation item receives a score according to the answer; 2 points if the answer is ‘yes’, 1 point if the answer is ‘partial’, or 0 points if the answer is ‘no’. If any analysis cannot be completed, there is a N/A option (not applicable). The evaluation is divided into the following phases: Total sum = (number of “yes” * 2) + (number of “partials” * 1), possible total sum = 28 − (number of “N/A” * 2) and Score summarized: total sum/possible total sum. The final percentage indicates the quality and the risk of bias for each study. In this review in the presence of any disagreement, a third reviewer (AMAS) evaluated the studies.

## 3. Results

### 3.1. Flow of Included Studies

**Figure 1:**
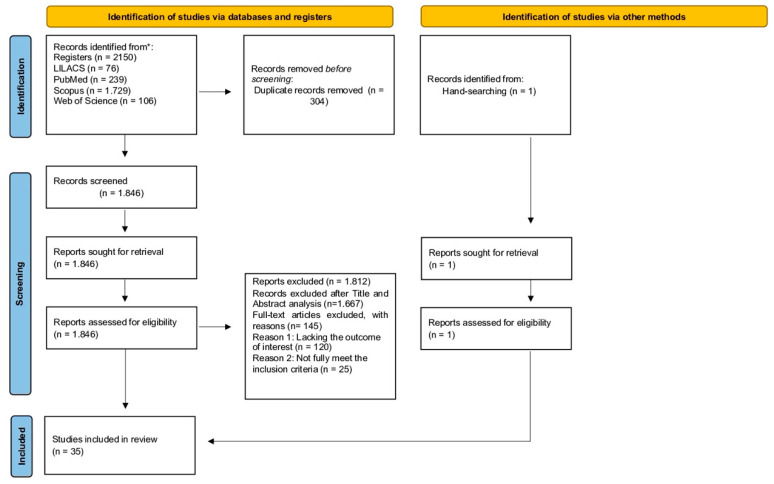
Flow diagram systematic search and review process.

Initially, 2150 studies were retrieved in the initial database search, 76 in LILACS, 106 in Web of Science, 1729 in Scopus, and 239 in PubMed, of which 304 were excluded because they were duplicates. After removing duplicates, 1846 studies remained and were screened by reading the titles and abstracts, of which 1667 were excluded. Subsequently, the reviewers read the full texts of the remaining 179 articles, of which 145 were ineligible because (i) they lacked the outcome of interest (n = 120) and/or (ii) they did not fully meet the inclusion criteria (n = 25). Thus, the electronic search generated 34 studies (Step 1), and 1 study was included via a manual search (Step 2). As a result, 35 articles were included in this review, as detailed in the PRISMA shown in Figure 1.

### 3.2. Description of the Studies

The selected studies were published from 1987 to 2019 and used quantitative (n = 24) and qualitative (n = 11) methodologies. Five studies were published before the launch of the GPELF (1997), and 30 studies were published after the launch.

All quantitative articles included in this review are described in Table 1. The selected studies covered different locations in the Americas: Brazil (n = 21), Dominican Republic (n = 5), Haiti (n = 6), and Guyana (n = 3) of which 14 concerned male urogenital disease (hydrocele and lymph scrotum) and 29 covered the topic of lymphoedemas (ADLA and elephantiasis).

Regarding male urogenital disease, 3 of the publications identified were in relation to pathogenesis, 14 were in relation to epidemiology, 3 were in relation to treatment, and 1 was in relation to the repercussions of those clinical manifestations. Regarding lymphoedema, 3 studies addressed the issue of pathogenesis, 13 addressed epidemiology, 8 addressed treatment, and 9 addressed the repercussions of this clinical manifestation.

In the Americas, groups of researchers from the Dominican Republic [8,9,10,11], Guyana [12,13], Haiti [14,15], and Brazil [16,17,18] published a few qualitative studies. They addressed the repercussions of those clinical manifestations, such as quality of life; economic and emotional impact; impact on activities; social support; stigma, and treatment with healthcare providers, beliefs, and traditional practices (Table 2).

**Table 1:** Details of data extracted from quantitative studies.

Author/Year	Country	Kind of Study	Population	Objective	Number of Morbidity Cases				Outcomes
					Elephantiasis	Hydrocele	Lymphoedema	Others	
Dreyer et al. (1987) [19]	Brazil	Quantitative	900	Describe the Filariasis Program created at CPqAM (Aggeu Magalhães Research Center, Recife-PE) with the installation of a specialized outpatient clinic	12	15	10	29—Tropical eosinophilia; 19—Lymphadenopathy; 10—Chyluria; 2—Chylocele; 1—Penis swelling	Identification of areas of infection foci for action
Addiss et al. (1995) [20]	Haiti	Quantitative	138	Assessment of filaria-specific immunological changes and association with disease progression	NA	62	64	NA	No association was found between antigen status and lower limb edema severity
Albuquerque et al. (1995) [21]	Brazil	Quantitative	3213	Observe the epidemiological characteristics of the disease before carrying out control protocols	9	63	51	392—Adenites; 35—Epididymo-orchites; 20—Adenolyphagitis; 6—Heamaturia; 5—Tropical pulmonary eosinophilia; 3—Chyluria	High prevalence of microfilaremia, low density of filariasis, and relatively low prevalence of filarial disease compared to endemicity
Norões et al. (1996) [22]	Brazil	Quantitative	15	Establish the prevalence and dilation of lymphatic vessels in the spermatic cord in individuals with *W. bancrofti*	NA	11	NA	NA	Lymphatic vessel dilatation was seen in all individuals with *W. bancrofti*
Braga et al. (1997) [23]	Brazil	Quantitative	967	Characterize the clinical and epidemiological profile of the disease in children and adolescents in an endemic location	0	10	1	4—Orchiepididymitis; 2—Adenolymphangitis	The parasitological survey attested to a large participation of children in the microfilaremic group
Vincent et al. (1998) [24]	Dominican Republic	Quantitative	32	Evaluate changes in anti-streptococcal antibodies diagnosed by anti-streptolysin and anti-DNAase tests in individuals with recurrent erysipelas	NA	NA	15	NA	Recurrent streptococcal invasion can generate expansion of lymphoedema and elephantiasis in individuals with chronic LF
Dreyer et al. (1999) A [25]	Brazil	Quantitative	600	Identification of AFL and ADLA triggers in people residing in an area endemic for LF	NA	NA	NA	582—Acute Dermatolymphangioadenitis; 18—Acute Filarial Lymphangitis	Most attacks of acute lymphangitis in areas where LF is endemic are not caused by filarial worms but by other infectious pathogens
Dreyer et al. (1999) B [26]	Brazil	Quantitative	273	To identify by ultrasonography, in a pediatric population, live adult worms in carriers of microfilariae and amicrofilaremic individuals	NA	NA	NA	11—Filaria dance sing (FDS)	FDS was detected in 11 children
Dreyer et al. (2002) [27]	Brazil	Quantitative	80	Determine the extent to which lymphatic dilatation occurs in the presence of adult *W. bancrofti*	NA	NA	NA	107—adult worm nests	Lymphatic vessel dilatation progresses in the presence of adult *W. bancrofti*, the rate of progression is heterogeneous
Bonfim et al. (2003) [28]	Brazil	Quantitative	9.520	Conduct an epidemiological survey to characterize LF	9	120	256	11—milky urine	LF remains a public health problem in the locality, requiring control measures
Norões et al. (2003) [29]	Brazil	Quantitative	569	To determine the association between adult *W. bancrofti* death and scrotal nodules and acute hydrocele and evaluate the clinical course of these hydroceles	NA	49, 40 with acute hydrocele and 9 with chronic hydrocele	NA	NA	Acute hydrocele occurs frequently following the death of adult the parasite and single episodes of scrotal nodule formation
Wilson et al. (2004) [30]	Haiti	Quantitative	91	Describe the histopathology of the skin at different stages of lymphoedema	NA	NA	91	NA	The results suggest that the improvement in morbidity associated with management has a histological origin
Fox et al. (2005) [31]	Haiti	Quantitative	192	To analyze the clinical picture and subclinical manifestations associated with *W. bancrofti* infection in children from an endemic location	NA	NA	NA	128—Interdigital lesions; 52—Inguinal lymph node pathology; 22—Severe interdigital lesions; 14—Crural lymph node pathology	Association between physical examination and circulating filarial antigen
Dreyer et al.(2006) A [32]	Brazil	Quantitative	119	Characterize the pattern of interdigital skin lesions in patients with lymphoedema of the lower limbs	NA	NA	119	ADLA	Interdigital skin lesions are a significant risk factor for ADLA and that people with lymphoedema in endemic localities
McPherson et al. (2006) [33]	Guyana	Quantitative	147	Assess the epidemiology of interdigital lesions of the feet in filarial lymphoedema	NA	NA	73	NA	Interdigital entry injuries can be risk factors for ADLA
Medeiros et al. (2006) [34]	Brazil	Quantitative	7650	Accomplish a population-based survey to assess the occurrence of LF	7	0	0	80—ADLA—Urogenital manifestations; 2—“milky urine”;	The findings demonstrate that the disease is still transmitted in the area
Freitas et al. (2008) [35]	Brazil	Quantitative	9465	Evaluate the epidemiological situation of LF in the city of Belém	0	3	1	18—ADLA; 2—“milky urine”	Results showed that there was an interruption in the transmission of the Belém focus
Aguiar-Santos et al. (2009) [36]	Brazil	Quantitative	6361	Provide data for the diagnosis and management of lymph scrotum cases	NA	3	NA	7—Lymph scrotum; 5—Lymphangiectasia; 2—Varicocele; 3—Nodule inside scrotum; 2—Testicular abnormality; 2—Epididymal abnormality; 1—Adenopathy	There is no consensus between the management methodologies for the different urogenital manifestations
Norões et al. (2009) [37]	Brazil	Quantitative	340	To analyze intrascrotal nodules in adult men as a marker of filarial granuloma in an endemic location	NA	1	NA	340—nodules; 1—adult worm nest with FDS signal	The results demonstrate great specificity of intrascrotal nodules with granuloma in endemic area residents
Norões et al. (2010) [38]	Brazil	Quantitative	1186	Analyze the occurrence of lymphangiectasia in the scrotal content, morphology, and consistency of the testicles and, recurrence of hydrocele using the complete excision of the tunica vaginalis in patients with hydrocele that	NA	968	NA	NA	Fluid from ruptured lymph vessels is an important component of filarial hydrocele, the authors advise complete excision of the hydrocele sac and, when identified, dilated lymph vessels with leakage or prone to leakage should be sutured or excised
Rocha et al. (2010) [39]	Brazil	Quantitative	10.021	Present the actions of the Lymphatic Filariasis Elimination Program in the city of Olinda-PE	15	158	15	184—ADLA; 2—Chyluria	Recommendations for evaluation and follow-up the impact of these actions of the Lymphatic Filariasis Elimination Program in the city of Olinda-PE
Netto et al. (2016) [40]	Brazil	Quantitative	23.673	Describe the prevalence of morbidity and its correlation with filarial infection	0	188	17	519—ADLA; 17—Chyluria	The findings indicate an association between reported clinical status and the rate of infection among people living in a low endemic area
Santana et al. (2016) [41]	Brazil	Quantitative	35	Describe the profile of patients with lymphedema who were subjected to Complex Decongestive Therapy	NA	NA	35	NA	The findings provide broad knowledge about the characteristics of individuals with lymphoedema who were subjected to Complex Decongestive Therapy
Soares et al. (2016) [42]	Brazil	Quantitative	30	Assess the effectiveness of Complex Decongestive Therapy using alternative inputs and its impact on the quality of life of individuals with lymphedema	NA	NA	30	NA	The methodology was considered efficient in reducing lymphoedema, with positive impacts on the quality of life of individuals

NA—Not Applicable.

**Table 2:** Details of data extracted from qualitative studies.

Author/Year	Country	Kind of Study	Population	Objective	Number of Morbidity Cases				Outcomes
					Elephantiasis	Hydrocele	Lymphoedema	Others	
Coreil et al. (1998) [15]	Haiti	Qualitative	29	Describe the impact of lymphoedema on women’s lives	29	NA	29	NA	Morbidity interferes socioeconomically in women’s lives
McPherson et al. (2003) [12]	Guyana	Qualitative	14	Describe, in individuals with lymphoedema, the impact on quality of life after inclusion of a hygiene and skin care regimen	NA	NA	14	NA	The care regimen was found to be effective in improving the quality of life of individuals with lymphoedema
Coreil et al. (2006) [14]	Haiti	Qualitative	Five support groups	Assess the process of indigenization in support groups for women with filarial morbidity	NA	NA	NA	NA	Creation of support groups with various activities focused on more technical terms related to LF and also on broader themes, such as religion, spirituality, handicrafts, and others
Dreyer et al. (2006) B [18]	Brazil	Qualitative	NA	Define the “Hope Club”, demonstrating its functioning and maintenance pillars	NA	NA	NA	NA	Report the experiences lived in “Hope Club”
Person et al. (2006) [8]	Dominican Republic	Qualitative	28	To observe health beliefs and self-care habits in women with lymphoedema	NA	NA	28	NA	The findings indicate that family, friends, and cultural habits influence the disease model
Person et al. (2007) A [10]	Dominican Republic	Qualitative	28	Understanding the psychosocial and health consequences associated with leg lymphoedema among women	NA	NA	28	NA	The women’s quality of life varied depending on changes in their health status, but the physical limitations were not always related to the severity of the symptoms
Person et al. (2007) B [9]	Dominican Republic	Qualitative	28	Observe the social connection among women with filarial lymphoedema	NA	NA	28	NA	Social disconnection can increase the negative effects of living with this morbidity
Person et al. (2009) [11]	Dominican Republic	Qualitative	104	Investigate women with lymphoedema who suffer from disease-related stigma	NA	NA	104	NA	Women described that they suffer from criticism and are isolated by the community, health professionals, and even by friends and relatives, in addition to being constantly denied access to education and work
Tyrell (2013) [13]	Guyana	Qualitative	100	To analyze the socioeconomic impact of LF in Guyana	NA	NA	NA	NA	Patients with chronic LF face significant impacts related to emotional and financial issues
Hettrick et al. (2017) [43]	Haiti	Qualitative	NA	Describe an action plan for people with filarial morbidity in Haiti	NA	NA	NA	NA	The use of appropriate management action plans have significant results in the quality of life of individuals with morbidity
Pedrosa et al. (2019) [16]	Brazil	Qualitative	25	To analyze the influence of unilateral lower limb lymphoedema on functionality and quality of life	NA	NA	25	NA	Individuals with lower unilateral lymphoedema suffer negative impacts on quality of life

NA—Not Applicable.

### 3.3. Quality Evaluation Criteria

The scores of the papers ranged from 36% to 100% for quantitative methodologies and 40% to 80% for qualitative methodologies. The values of <50% indicated studies that included less information or presented with incomplete data. Scores ranging from 64% to 88% were applied to articles that had the most complete data. In addition, scores from 90% to 100% were applied to those with the best clarity of information. Additional File 2 (Tables S1 and S2) present the scores in detail from the quantitative and qualitative studies, respectively.

## 4. Discussion

More focus on the second pillar of the GPELF program, morbidity management, is increasingly required. Valid and reliable information regarding the number of people affected in each of the most frequent clinical expressions, the treatment approach instituted, the structure of care services offered, responses to treatments, disability and the impact of disease, and patient and community needs are important in the assessment and planning in endemic areas to ensure the best management for the prevention and alleviation of lymphatic filariasis-related disability. As a strategy for use in structuring the assistance of filarial morbidity, the GPELF suggests the identification of the number of individuals affected by the disease in areas where MDA is implemented.

This review identified insufficient data regarding the number of cases of morbidity in endemic countries. No population surveys were carried out in any of the countries studied, which is indicative of under-reporting, which is a trend that had already been highlighted in other endemic areas [4]. There are also gaps in studies with a robust methodology that can be “replicable” for treatment assessment, such as physical therapy for lymphedema treatment and the surgical technique used for the treatment of hydrocele. A small number of publications related to properly structured operational and implementation investigations, including community-based and applied research, which is essential for building a solid foundation on which effective interventions can be designed and carried out as suggested by WHO [4].

In this review, two major clinical manifestations were addressed: male urogenital disease (hydrocele and lymph scrotum) and lymphoedema (including ADLA and elephantiasis). Due to the non-uniformity of the methodologies used in the addressed studies, comparison of the data was limited and, therefore, we chose to present the information in similar topics referring to these manifestations.

### 4.1. Male Urogenital Disease: Hydrocele and Lymph Scrotum

Although hydrocele and lymph scrotum exert a significant impact on public health and men’s lives in endemic areas, many gaps in the knowledge regarding these issues mean that certain topics still remain unclear, such as ideal management, intervention costs and their complications, and economic, social, emotional, and sexual repercussions.

### 4.2. Pathogenesis

The biggest contributions related to the topic of male urogenital disease manifestations came from Brazilian authors and included: (i) longitudinal ultrasonographic measurements of the intrascrotal lymphatic vessel diameter at the site of living adult *W. bancrofti* observed that diameter increased in a high percentage of adult worm nests [27]; (ii) no significant difference was observed in the rate of scrotal lymphatic vessel dilatation among men who were treated or not treated with antifilarial drugs (diethylcarbamazine—DEC) [29]; (iii) the identification rate of hydrocele was higher in men who received DEC than in untreated men, and during the follow-up period, this manifestation did not resolve within 18 months or continued to increase in size [29]; (iv) an important association between filarial infection and the inadequate surgical and clinical management of hydrocele was identified as a risk factor for lymph scrotum [36]. All of this information corroborates the findings of previous studies that concerned residents of areas where filariasis is endemic that show that the primary lesion in these populations is not an obstruction but lymphatic vessel dilatation [44,45]; other studies have demonstrated that filarial hydrocele is triggered by the death of an adult worm, which produces an inflammatory nodule that occludes the lymphatic vessel [46,47,48]. Regarding lymph scrotum, a dermatological manifestation of pathological lymphatic drainage, which is an unusual urological clinical presentation of LF, may be identified following surgery for chylocele or hydrocele [49].

### 4.3. Epidemiology

There is no real estimate of the number of men affected by hydrocele in endemic areas, but there is a strong association of its prevalence with that of microfilaremia (MF), and the prevalence of hydrocele increases with age [50,51]. Studies in Haiti show that in men with hydrocele, there is a high percentage of active *W. bancrofti* infection [20]. Additionally, these studies show that in areas where nearly half of the population has LF, 25% of the males have genital lymphoedema and scrotal damage due to the disease (hydrocele) and that this is often hidden and unreported [43].

In Brazil, a 5.5% prevalence of hydrocele was found among 1177 individuals, 26% of the subjects with hydrocele were MF carriers [21], and in children, the prevalence of infection was 6.4%; 3.8% of children in the study were identified as having hydrocele, with a predominance in the age group of 10–14 years [23]. Ultrasonographic studies were important in identifying hydrocele in microfilaremic men [22] and children [26]. Some studies presented percentage rates of participants who reported a complaint ranging from 1.1 to 1.57% [26,28,34,39,40], and another study showed that only isolated cases were identified [35].

### 4.4. Repercussions of Clinic Manifestation

Aguiar-Santos et al., 2009 [36], identified that in all evaluated cases of lymph scrotum, there was a report of the occurrence of ADLA due to secondary bacterial infection that was constant and repeated. This medical issue causes the continuous drainage of milky secretion; affected individuals are often prevented from engaging in work or leisure activities, changes may occur to their social interactions, and they may have issues regarding sexual intercourse. They may also experience symptoms of depression. In this review, it was observed that a small number of studies exist beyond the biological scope that has focused on the assessment of the repercussions of male urogenital manifestations of FL in the social, emotional, and sexual spheres in the affected population.

### 4.5. Treatment

It is emphasized that the treatment with DEC for individuals when they present microfilaremia may be of fundamental importance for the control of transmission, highlighting the fact that these individuals continue to live in endemic areas, and are at risk of reinfection [36]. A retrospective study in Brazil emphasized that the best way to monitor the effectiveness of antifilarial drugs is the combined use of ultrasound and physical examination to monitor the development of scrotal nodules [37].

Regarding hydrocele surgery, a selected paper concluded that with the intent to avoid hydrocele recurrence and testicular damage, a specific surgical technique that involves the complete excision of the tunica vaginalis and meticulous attention to cauterization of the edges and suturing offers the best cosmetic and functional result with a lower risk of lymph scrotum [38]. Once the lymph scrotum is identified, the approach to this morbid condition is based on conservative measures that avoid its progression. These measures consist of local hygiene care, in the same manner as that proposed for lymphoedema in the limbs, and antibiotic prophylaxis using benzathine penicillin. However, in some cases, surgical reconstruction has been shown to be efficient, considering that this chronic disease is of an irreversible nature [36].

### 4.6. Lymphoedema—ADLA and Elephantiasis

One of the most distressing clinical presentations of lymphatic dysfunction in LF in endemic areas is advanced lymphoedema or elephantiasis which for reasons that are poorly understood, affect women more frequently than men in many parts of the world, including in the Americas [15,20]. Lymphoedema is a chronic condition that is not curable at present, but may be alleviated by the appropriate management of ADLA; if ignored, it can progress and become difficult to manage.

### 4.7. Pathogenesis

Dreyer et al., 1999 [25], reported two distinct acute syndromes accompanied by lymphangitis; one is called acute filarial lymphangitis (AFL), which is caused by the death of adult worms, and usually, it is asymptomatic or has a mild clinical course and rarely causes residual lymphoedema. The second syndrome is called ADLA, which is not caused by filarial worms per se, but probably results from a secondary bacterial infection. The majority of patients with ADLA reported experiencing a recurrence of the attacks which are considered to be the main risk factor for the development of lymphoedema and elephantiasis, as previously described [52,53] There is clinical evidence that AFL precedes the onset of ADLA in a portion of the population, suggesting that LF is a risk factor for secondary infections [32].

Studies carried out in Haiti and Guyana showed that severe interdigital lesions were associated with laboratory evidence of filarial infection [31] and that the number of lesions was the strongest predictor of the frequency of ADLA [33]. Additionally, studies in the Dominican Republic concluded that about half of the acute episodes identified were caused by, or associated with, infections with group A hemolytic streptococci [24].

### 4.8. Epidemiology

Few prevalence studies that were representative of the population at risk in endemic areas of the Americas were identified; some studies gave a small estimate of the number of those affected and their significance as a public health problem [19,21,23,28,32]. It was observed that there is an underestimation of the number of patients with lymphoedema, which is largely underestimated in areas still endemic, as well as in those areas that have already achieved transmission disruption.

In Brazil, a variation in the frequency of lymphoedema was also observed according to the prevalence of filarial infection in the study area and characteristics of the population studied (age and sex), in addition to the method of collection, whether by clinical examination or reported complaint. In general, the frequency of lymphoedema is higher in women, it increases with age, and in surveys that concerned reported complaints, ADLA citation is quite high [21,23,28,34,35,39,40]. ADLA incidence was associated with the lymphoedema stage, and the number of interdigital skin lesions detected by the examining physician [32].

In Haiti, in specific hyperendemic zones, estimates suggest that even 5% of the Haitian population suffers from lymphoedema associated with LF. Many women and girls suffer from leg elephantiasis [43].

In patients with lymphoedema, a high occurrence of comorbidities was also identified, with the most frequent being obesity, hypertension, and diabetes [41]. These data suggest that care services for patients with filarial morbidity include specialized care that also aims to address these problems [54].

### 4.9. Repercussions of Clinic Manifestation

Our findings regarding the repercussions of lymphoedema are all qualitative studies published regarding endemic countries after the launch of the GPELF. No reports were identified in the areas of America that have already eliminated filariasis (Trinidad, Suriname, and Costa Rica).

Several publications indicate that morbidity caused by LF has dramatic social, economic, and emotional consequences, particularly for women, who are over 10 times more likely to have elephantiasis of the leg than men [55,56,57,58,59]. Having knowledge of the consequences of this morbidity in these various aspects will support countries in structuring assistance services to provide care in a holistic way, as is the goal established in the GPELF.

Qualitative studies carried out in the Dominican Republic and Guyana have made important contributions towards investigating how women from different cultures with lymphoedema experience stigma and its consequences [11]. Some women felt that speaking about these practices would be uncharitable and that the burden they faced was a test from God and described a cultural practice of not talking about or belittling someone with a disease or disability because to do so would put one at risk of getting the same condition. For some women, it was the quantity or burden of stigma experiences that meant they had ineffective management or coping capabilities that created distressing personal consequences. Poverty, poor access to healthcare resources, limited education, and diminished social support challenged the coping strategies of many women and exacerbated the negative consequences of lymphoedema-related stigma [8,9,10,11].

For these women, indigenous healers have influence over the physical, mental, spiritual, and supernatural properties of illness for initial care, and only when indigenous treatments proved to be ineffectual did they seek care from trained healthcare providers [8]. In other endemic areas not located in the Americas, traditional practices for lymphoedema include herbal preparations which are smeared on the affected limb, scarification or cutting the skin, analgesics bought from local drug peddlers [60,61,62], and inappropriate treatments, such as diuretic therapy [63].

Women described a spectrum of consequences associated with their lymphoedema, but physical, functional, and psychological limitations were not always associated with the severity of lymphoedema [10]. The confluence of chronic and acute illness with the severity of lymphoedema leads women to rely on others for social support. Women described complications regarding aging, disability, reduced social networks, and the inability to adhere to cultural scripts as contributors to disrupted social connectedness. Most women resorted to self-prescribing injectable, oral, or topical antibiotics along with oral analgesics without medical supervision [9].

An intervention study was conducted in Guyana that concerned patients with lymphoedema that was clinically confirmed and classified by the Dreyer scale [64] The patients were interviewed regarding their disease history and current acute attack rates, they also answered the questionnaire about current knowledge, attitudes, and practices (KAP) and completed a Dermatology Life Quality Index (DLQI). After this, interventions were put in place, which included education and training of a local nurse, individual patient education, and access to appropriate treatments. This low-cost intervention study had a significant impact on the quality of life of patients with a reduction in the number of acute attacks, leading to the increased ability to work and perform activities of daily living [12].

Tyrell and colleagues, 2013 [13] explained that there is a necessity for the establishment of support groups where issues and problems can be discussed in an atmosphere of empathetic understanding with the use of social networks and services, such as Skype and Google. The number of lymphoedema and hydrocele patients in lymphatic filariasis endemic communities are usually under-reported, which consequently affects their management. Therefore, an innovative tool can give accurate information on morbidity cases in real-time in Ghana and can also reduce the under-reporting of cases is need [65].

In a community located about 30 km west of Port-au-Prince (Haiti) where the prevalence of microfilaremia was 33% and about one-half of the population was infected with *W. bancrofti*, researchers described the process of indigenization within peer support groups for Haitian women living with chronic physical impairment of LF. Five support groups established in a coastal community were studied over a period of 3 years to understand the adaptation of the Western illness support group model to the local cultural milieu. Unlike most support groups in affluent settings, the Haitian women showed minimal interest in talking about illness-related issues. The groups developed a distinctly Haitian style characterized by an emphasis on religion and spirituality, artistic and expressive components, and the acquisition of practical skills that offer income-generating opportunities. Members directed the greatest energy toward developing microenterprise activities [14]. In another study in Leogane, Haiti, researchers investigated the ethnographic context of filarial elephantiasis among women and focused on explanatory models of the illness, the impact of the disease on women’s lives, and the difficulties patients experienced in following a therapeutic regimen provided at a local hospital [15].

The results indicate that traditional understanding and treatment for the disease are prevalent in the community. These studies show that the pattern of adaptation is discussed in terms of indigenous traditions of mutual aid in rural Haiti, the compelling material needs of families living in stark poverty, and the ongoing challenge of coping with political and economic insecurity. Women’s lives are substantially burdened both socially and economically by the physical impairment of elephantiasis, most notably by the loss of income due to restrictions on mobility. The degree of social discrimination encountered varies by the timing of the onset of symptoms in one’s life course. Difficulties encountered with the physical therapy regimen included the maintenance of the compressive bandage and the availability of suitable footwear.

Three studies were carried out in Brazil focusing on sequelae in patients with chronic lymphatic filariasis. Dreyer, Norões, and Mattos, 2006 [18], developed a care program that could equip lymphoedema patients with the skills, motivation, and enthusiasm to sustain an effective, low-cost, and convenient self-care to prevent episodes of ADLA and milky urine in the case of chyluria carriers. This innovative care approach was called Hope Clubs. For people with elephantiasis, recovery of full “citizenship” and human dignity can be a powerful strategy for overcoming prejudice and stimulating socially responsible action. The synergy—and even magic—that is created when patients come together to share their lives and experiences in Hope Club meetings creates the possibility for the multiplication of knowledge. Hope Clubs are a powerful instrument for generating action for the social inclusion of people with elephantiasis [17]. In a group of 25 patients with lymphoedema, quality of life was compromised in all domains of the SF-36; they were principally affected in the domains of functional capacity (45.4 ± 25.9), emotional aspects (36.0 ± 42.9), and limitations due to physical aspects (25.0 ± 31.4). Those studies point to a necessity for the establishment of support groups where issues and problems can be discussed in an atmosphere of empathetic understanding [13].

### 4.10. Treatment

There is no proven drug treatment for lymphoedema. Management aims to reduce or delay the progression of swelling and prevent associated infection [63].

In Brazil, a study highlighted some points regarding the treatment of ADLA and LFA. The use of DEC, an antifilarial drug, did not stop or shorten the duration of acute episodes of either ADLA or LFA and did not prevent the recurrence of ADLA, nor did it stop disease progression, although the majority of the patients with elephantiasis who were referred had been previously treated with up to six full courses of DEC [25]. On the other hand, there is evidence that persistent local hygiene and the cure and prevention of entry lesions with antiseptics and topical antibiotic/antimycotic salves can stop ongoing attacks and completely prevent their recurrence [66]. In advanced cases, it may be necessary to combine such topical treatments with antibiotic prophylaxis to achieve this goal. Additionally, to prevent the recurrence of ADLA and progression to elephantiasis, it is essential that patients with lymphoedema are able to promptly identify and treat interdigital skin lesions. Studies indicate that among patients coming to the clinic for the first time, very few were aware that they had interdigital skin lesions, or if they were aware, they did not recognize the lesions as abnormal [32]. Physiotherapy using the edema reduction technique called Complex Decongestive Therapy (CDT), which aims to promote lymphatic return and a reduction in frequent pain, was shown to be effective in reducing and controlling lymphoedema and had a positive impact in some studies, causing participants in intervention groups to provide more positive answers to questionnaires regarding quality of life in physical and environmental domains [41,42].

In the Dominican Republic, in a study that concerned the response of anti-streptolysin 0 titer and anti-DNAase B antibodies in the 28 erysipelas cases enrolled, benzathine penicillin was highly used and intramuscular injection usually brought an almost dramatic relief for the patients [24]. In Guyana, a study analyzed the impact on the quality of life that education and the introduction of organized care had on lymphoedema patients after they were individually educated on lymphatic filariasis, given appropriate advice on how to manage their lymphoedema, educated on the importance of hygiene, skin care, and elevation, and taught simple exercises to encourage lymph drainage. Treatment was given from a formulary of antibiotics (Penicillin) (in the case of a current acute attack), antiseptics (potassium permanganate), and topical creams (Bacitracin ointment and Miconazole 2%). Each patient was given a tube of a topical antibacterial and antifungal cream if clinically indicated, and a patient education leaflet. The Dermatology Life Quality Index, a questionnaire that is an accurate way of assessing the effect skin disease has on quality of life was repeated 1 year later and improved for all patients, and the number of reported acute attacks was reduced. This study shows that in this community, with only a very limited input of time and financial resources, a change/introduction in the treatment regime can have a beneficial impact on patients with lymphoedema within a year [12].

In Haiti, the effect of basic lymphoedema management (hygiene, skin care, and lower limb movement and elevation) was investigated via biopsy specimens collected during the follow-up of 27 patients. Biopsies showed reductions in perivascular mononuclear infiltrate in the superficial dermis, perivascular fibrosis in the deep dermis, and periadnexal mononuclear infiltrate suggesting clinical improvement [30]. In 2012, a morbidity management program was established based on a series of training programs and provided the requisite knowledge to appreciate the pathophysiology associated with LF, along with didactic and hands-on training involving modified protocols encompassing Complete Decongestive Therapy and basic skin and wound care principles. The technicians taught the patients proper skin care and hygiene and provided modified protocols involving manual lymph drainage, diaphragmatic breathing, compression, patient education, and basic wound care. Overall, the results were impressive including, but not limited to significant reductions in limb volume; improved skin integrity; independence with self-care; enhanced quality of life; improvements in functional abilities and activities of daily living; the ability to return to work; and reduced perceptions of social stigma [43].

In this review study, there were limitations in comparing the results found, particularly due to the variety of methodologies used by researchers. Despite the increase in the number of studies after the launch of the GPEFL, it was observed that the information contained in the publications is still insufficient to identify the number of people affected, the effectiveness of treatments, and the structuring of services to accommodate the cases identified in the Americas.

## 5. Conclusions

In this review, we sought to analyze publications related to the handling of filarial morbidity, particularly male urogenital manifestations and lymphoedema, as the most frequent sequelae observed in endemic areas. The findings were limited to publications from American countries where these diseases are still considered endemic: Brazil, Guyana, Haiti, and the Dominican Republic, and it also aimed to identify the conduction of the GPELF morbidity pillar in these countries, its epidemiology, treatment used, economic and social consequences, psychological and care structures already in place, as there are gaps in this knowledge. This knowledge is essential if the WHO Global Control of Lymphatic Filariasis Validation is to be achieved. Current burden estimates are limited in reliability because of the paucity of survey data available on which to base estimates of the total number of cases. In order to develop more robust estimates of the burden of filarial morbidity, there needs to be a coordinated effort to conduct population-based surveys.

Thus, to prevent suffering and disability in those who already have chronic manifestations of lymphatic filariasis, as recommended by the GPELF, it is necessary that countries of the Americas, through a coordinated effort from health services and researchers, develop strategies that aim to address the pillar of morbidity through the implementation and maintenance of services intended for this purpose.

## Data Availability

The data presented in this study are available in the present article and its Supplementary Materials reported above.

## Supplementary Materials

The following are available online at https://www.mdpi.com/article/10.3390/ijerph19010316/s1, Table S1: Quality evaluation of the included quantitative studies, Table S2: Quality evaluation of the included qualitative studies.
