# Supplementary material for: Lymphatic Filariasis: A Systematic Review on Morbidity and Its Repercussions in Countries in the Americas
Source: Int J Environ Res Public Health. 2021 Dec 28;19(1):316. doi: 10.3390/ijerph19010316 (PMC8751179; doi:10.3390/ijerph19010316)
Supplement: Supplementary file 1 [file ijerph-19-00316-s001.zip › Table S1.pdf]

Table S1: Quality evaluation of the included quantitative studies.

Criteria	Studies							
	Dreyer *et al.* 1987 [50]	Addiss *et al*. 1995 [30]	Albuquerque *et al.*, 1995 [32]	Norões *et al*., 1996 [34]	Braga *et al*., 1997 [33]	Vincent *et al*., 1998 [49]	Dreyer *et al*., 1999 A [43]	Dreyer *et al*., 1999 B [35]
Question/objective sufficiently described?	0	2	2	2	2	2	1	2
Study design evident and appropriate?	1	2	2	2	2	2	2	2
Method of subject/comparison group selection or source of information/input variables described and appropriate?	1	2	2	1	1	1	1	1
Subject (and comparison group, if applicable) characteristics sufficiently described?	1	1	2	1	1	1	1	1
If interventional and random allocation was possible, was it described?	N/A	N/A	N/A	N/A	N/A	N/A	N/A	N/A
If interventional and blinding of investigators was possible, was it reported?	N/A	N/A	N/A	N/A	N/A	N/A	N/A	N/A
If interventional and blinding of subjects was possible, was it reported?	N/A	N/A	N/A	N/A	N/A	N/A	N/A	N/A
Outcome and (if applicable) exposure measure(s) well defined and robust to measurement / misclassification bias?Means of assessment reported?	1	1	1	1	1	1	1	1
Sample size appropriate?	1	1	2	1	2	1	1	2
Analytic methods described/justified and appropriate?	0	2	2	1	2	2	0	2
Some estimate of variance is reported for the main results?	1	1	1	1	1	2	1	1
Controlled for confounding?	0	0	0	0	0	0	0	0
Results reported in sufficient detail?	1	2	2	2	2	2	1	2
Conclusions supported by the results?	1	2	2	2	2	1	2	1
**Maximum points**	22	22	22	22	22	22	22	22
**Total points**	08	16	18	14	16	15	13	15
**Summary score (%)**	36	73	82	64	73	68	59	68

0 if the response is ‘no’; 1 if the response is ‘partial’; 2 if the response is ‘yes’; followed by N/A if not applicable.

Table S1: Quality evaluation of the included quantitative studies.

**Criteria**	**Studies**							
	Dreyer *et al*., 2002 [21]	Bonfim *et al*., 2003 [36]	Norões *et al*., 2003 [25]	Wilson *et al*. 2004 [66]	Fox *et al*. 2005 [47]	Dreyer *et al*. 2006 A [46]	McPherson *et al.* 2006 [48]	Medeiros et al. 2006 [37]
Question/objective sufficiently described?	1	2	2	2	2	2	2	2
Study design evident and appropriate?	2	2	2	1	2	1	1	2
Method of subject/comparison group selection or source of information/input variables described and appropriate?	2	2	1	2	1	1	0	2
Subject (and comparison group, if applicable) characteristics sufficiently described?	1	1	1	1	2	2	0	1
If interventional and random allocation was possible, was it described?	N/A	N/A	N/A	N/A	N/A	N/A	N/A	N/A
If interventional and blinding of investigators was possible, was it reported?	N/A	N/A	N/A	N/A	N/A	N/A	0	0
If interventional and blinding of subjects was possible, was it reported?	N/A	N/A	N/A	N/A	N/A	N/A	N/A	N/A
Outcome and (if applicable) exposure measure(s) well defined and robust to measurement / misclassification bias?Means of assessment reported?	1	1	1	2	2	2	1	2
Sample size appropriate?	2	2	1	2	2	2	1	2
Analytic methods described/justified and appropriate?	2	1	2	2	2	2	2	1
Some estimate of variance is reported for the main results?	1	2	2	1	2	2	2	2
Controlled for confounding?	0	0	0	N/A	N/A	N/A	0	N/A
Results reported in sufficient detail?	2	2	2	2	2	2	1	2
Conclusions supported by the results?	2	2	2	2	2	2	2	2
**Maximum points**	22	22	22	20	20	20	24	22
**Total points**	16	17	17	17	19	18	12	18
**Summary score (%)**	73	77	77	85	95	90	50	82

0 if the response is ‘no’; 1 if the response is ‘partial’; 2 if the response is ‘yes’; followed by N/A if not applicable.

Table S1: Quality evaluation of the included quantitative studies.

Criteria	Studies							
	Freitas *et al.*, 2008 [40]	Aguiar-*Santos et al.*, 2009 [27]	Norões et al. 2009 [41]	Norões *et al.*, 2010 [42]	Rocha *et al.*, 2010 [39]	Netto *et al.*, 2016 [38]	Santana *et al.* 2016 [52]	Soares *et al.*, 2016 [65]
Question/objective sufficiently described?	2	2	2	2	2	2	2	2
Study design evident and appropriate?	1	2	2	2	1	1	2	2
Method of subject/comparison group selection or source of information/input variables described and appropriate?	2	2	1	2	1	2	2	2
Subject (and comparison group, if applicable) characteristics sufficiently described?	0	1	1	2	1	1	2	1
If interventional and random allocation was possible, was it described?	N/A	N/A	N/A	N/A	N/A	N/A	N/A	2
If interventional and blinding of investigators was possible, was it reported?	N/A	N/A	N/A	N/A	N/A	N/A	N/A	1
If interventional and blinding of subjects was possible, was it reported?	N/A	N/A	N/A	N/A	N/A	N/A	N/A	0
Outcome and (if applicable) exposure measure(s) well defined and robust to measurement / misclassification bias?Means of assessment reported?	1	2	2	2	2	2	1	2
Sample size appropriate?	2	2	2	2	1	2	1	2
Analytic methods described/justified and appropriate?	0	N/A	N/A	2	1	2	2	2
Some estimate of variance is reported for the main results?	0	N/A	N/A	2	1	2	2	1
Controlled for confounding?	N/A	N/A	N/A	N/A	0	N/A	N/A	0
Results reported in sufficient detail?	1	2	2	2	1	2	2	1
Conclusions supported by the results?	2	2	2	2	2	2	2	2
**Maximum points**	20	16	16	20	22	20	20	28
**Total points**	11	15	14	20	13	18	18	20
**Summary score (%)**	55	94	88	100	59	90	90	71

0 if the response is ‘no’; 1 if the response is ‘partial’; 2 if the response is ‘yes’; followed by N/A if not applicable.
