# Supplementary material for: Lymphatic Filariasis: A Systematic Review on Morbidity and Its Repercussions in Countries in the Americas
Source: Int J Environ Res Public Health. 2021 Dec 28;19(1):316. doi: 10.3390/ijerph19010316 (PMC8751179; doi:10.3390/ijerph19010316)
Supplement: Supplementary file 1 [file ijerph-19-00316-s001.zip › Table S2.pdf]

**Table S2:** Quality evaluation of the included qualitative studies.

| Criteria | Studies | | | | | |
|---|---|---|---|---|---|---|
| | Coreil *et al*., 1998 [15] | Mc Pherson *et al*., 2003 [12] | Coreil *et al*., 2006 [14] | Dreyer et al., 2006 B [18] | Person *et al*. 2006 [8] | Person *et al*. 2007 A [10] |
| Question/objective sufficiently described? | 2 | 2 | 2 | 1 | 2 | 2 |
| Study design evident and appropriate? | 2 | 2 | 2 | 1 | 1 | 2 |
| Context for the study clear? | 2 | 2 | 2 | 2 | 2 | 2 |
| Connection to a theoretical framework/wider body of knowledge? | 2 | 2 | 2 | 2 | 2 | 2 |
| Sampling strategy described, relevant and justified? | 2 | 1 | 1 | 0 | 2 | 1 |
| Data collection methods clearly described and systematic? | 1 | 2 | 1 | 0 | 2 | 2 |
| Data analysis clearly described and systematic? | 1 | 2 | 1 | 0 | 2 | 1 |
| Use of verification procedure(s) to establish credibility? | 1 | 0 | 1 | 0 | 0 | 0 |
| Conclusions supported by the results? | 1 | 2 | 2 | 2 | 2 | 2 |
| Reflexivity of the account? | 2 | 0 | 0 | 0 | 0 | 0 |
| **Maximum points** | 20 | 20 | 20 | 20 | 20 | 20 |
| **Total points** | 16 | 15 | 14 | 8 | 15 | 14 |
| **Summary score (%)** | 80 | 75 | 70 | 40 | 75 | 70 |

0 if the response is ‘no’; 1 if the response is ‘partial’; 2 if the response is ‘yes’ followed by N/A if not applicable.

**Table S2:** Quality evaluation of the included qualitative studies.

| Criteria | Person *et al*. 2007 B [9] | Person *et al*. 2009 [11] | Tyrell *et al*. 2013 [13] | Hettrick 2017 [31] | Pedrosa *et al*. 2019 [16] |
|---|---|---|---|---|---|
| | **Studies** | | | | |
| Question/objective sufficiently described? | 2 | 1 | 2 | 1 | 2 |
| Study design evident and appropriate? | 2 | 2 | 2 | 1 | 2 |
| Context for the study clear? | 2 | 2 | 2 | 2 | 2 |
| Connection to a theoretical framework/wider body of knowledge? | 2 | 2 | 2 | 2 | 2 |
| Sampling strategy described, relevant and justified? | 1 | 1 | 1 | 0 | 2 |
| Data collection methods clearly described and systematic? | 2 | 2 | 1 | 0 | 2 |
| Data analysis clearly described and systematic? | 1 | 1 | 2 | 0 | 2 |
| Use of verification procedure(s) to establish credibility? | 0 | 0 | 0 | 0 | 0 |
| Conclusions supported by the results? | 2 | 2 | 2 | 2 | 2 |
| Reflexivity of the account? | 0 | 0 | 0 | 0 | 0 |
| **Maximum points** | 20 | 20 | 20 | 20 | 20 |
| **Total points** | 14 | 13 | 14 | 8 | 16 |
| **Summary score (%)** | 70 | 65 | 70 | 40 | 80 |

0 if the response is ‘no’; 1 if the response is ‘partial’; 2 if the response is ‘yes’ followed by N/A if not applicable.
